# Hub genes of neutrophil extracellular traps in abdominal aortic aneurysm: a bioinformatics analysis

**DOI:** 10.1186/s41065-026-00663-0

**Published:** 2026-04-20

**Authors:** Jinxin Liu, Bixuan Yue, Zhiying Shen, Bo Yang, Yuxuan Ou, Xiaowu Wang

**Affiliations:** 1 Department of Cardiovascular Surgery, Zhujiang Hospital, Southern Medical University, Gongye Avenue, Haizhu District, Guangdong 510280 Guangzhou, China; 2 Department of Cardiovascular Medicine, Zhujiang Hospital, Southern Medical University, Guangzhou, Guangdong 510280 China; 3 Department of Orthopedics, Zhujiang Hospital, Southern Medical University, Guangzhou, Guangdong 510280 China

**Keywords:** Abdominal aortic aneurysm, Bioinformatics analysis, Inflammatory response, NETs, PADI4

## Abstract

**Purpose:**

This study aimed to identify the key genes involved in the development of AAA by various bioinformatics methods and explore their potential mechanisms.

**Methods:**

Feature genes were screened by machine learning and other methods, and ROC curves were used to verify the model performance in the internal and external validation sets. Cluster analysis and immune infiltration analysis were then performed, and two key genes were finally determined and experimentally verified. GSEA, GO and KEGG analyses were performed.

**Results:**

DEGs were identified by the “limma” package. DENETs were obtained by intersecting DEGs with NETs. GSEA, GO and KEGG analyses revealed that these genes were enriched in pathways such as cell response to lipopolysaccharide and chemokine signaling. Cluster analysis was performed to compare the expression differences of 25 DENETs between clusters, and immune infiltration analysis revealed immune dysregulation in AAA. Then, IL6 and PADI4 were finally identified as DENETs, and further GSEA analysis revealed that they were related to inflammation and immune response. In the wet experiment, IL6 and PADI4 finally showed expression trends consistent with the results of bioinformatics analysis.

**Conclusions:**

Our study identified IL6 and PADI4 as hub genes. They play an important role in promoting the development of AAA through inflammation, providing potential molecular targets for further treatment and intervention of AAA in the future.

**Supplementary Information:**

The online version contains supplementary material available at 10.1186/s41065-026-00663-0.

## Introduction

Abdominal aortic aneurysm (AAA) is a full-thickness segmental dilatation of the abdominal aorta, which is 50% larger than the normal artery diameter, and it is usually diagnosed based on a maximum aortic diameter of ≥ 30 mm on imaging [1]. Aneurysms are usually asymptomatic before rupture, and pulses may be felt in the abdominal region in a small number of patients [2]. As they grow bigger, symptoms such as abdominal and back pain may occur. Once rupture occurs, it can lead to massive abdominal bleeding, which may threaten life if not treated urgently [3]. The post-rupture mortality is about 85%-90% [4]. Aging (> 60 years), smoking, hypertension, and atherosclerosis are recognized risk factors that promote AAA progression [3–5]. The incidence of AAA in men is 2–4 times that in women of the same age, but women are at higher risk of AAA rupture [6, 7].

Ultrasound, computed tomography (CT), and magnetic resonance imaging (MRI) are the means for imaging diagnosis of AAA, and clinical treatments mainly include medication, open surgery, and endovascular aortic repair (EVAR) [8–10]. Nonetheless, the complication and mortality rates associated with surgery are still high, with a poor long-term prognosis. No reliable medication is available yet for restricting the AAA growth or rupture [11]. Consequently, searching for novel clinical biomarkers and drug targets is imperative for delaying the AAA progression in the future.

The pathology of AAA involves the interaction of endothelial cells, monocytes, macrophages, vascular smooth muscle cells (VSMCs), and immune cell infiltration, leading to inflammation-driven vascular wall degeneration and apoptosis [12–16]. In the case of inflammation, monocytes and neutrophils are recruited into the AAA microenvironment, resulting in cellular phenotypic change and activation of adaptive immune response [17]. Therefore, immune cells and inflammation are crucial players in AAA. Previously, it was found that neutrophil mediators, for instance, myeloperoxidase (MPO), matrix metalloproteinase (MMP), and neutrophil elastase (NE) can facilitate the AAA development through extracellular matrix (ECM) degradation and oxidative stress [18].

Neutrophils are continuously produced by bone marrow progenitors in the bone marrow [19], with mechanisms involving phagocytosis, degranulation, and cytokine production [20, 21]. Neutrophil extracellular traps (NETs), composed of histones, DNA, and granule proteins, are extracellular web-like structures formed by neutrophils in the context of pathogens [22, 23]. The activation and release of NETs is often termed NETosis [24]. Neutrophils can facilitate ECM degradation via matrix metalloproteinase-9 (MMP9) in AAA [25]. NETs can capture and kill pathogens, regulate immune responses, and participate in inflammatory processes [26]. However, excessive or abnormal formation of NETs may cause tissue damage and diseases such as autoimmune diseases and thrombosis [22]. NETs may exert a pivotal role in the AAA pathogenesis. Specifically, NETs will enhance inflammatory responses and thus lead to weakening and degenerative lesions of the arterial wall, worsening AAA [17]. In addition, NETs can enhance the interleukin-6 (IL6) and pro-IL1β transcription in macrophages, induce Th17 cell differentiation, and recruit more inflammatory cells [27]. With a web-like structure, NETs allow blood cells to accumulate in the aorta, ultimately leading to thrombosis and vascular occlusion [23]. However, the targets of NETs in AAA are rarely reported, and their mechanism in AAA remains to be further investigated.

By comparing differences between patients and healthy individuals, bioinformatics is a useful technique for discovering critical molecules and studying the underlying molecular mechanisms of disease. In the current study, bioinformatics analysis was executed on the hub genes of NETs in the development of AAA, and the role of two novel clinical biomarkers IL6 and PADI4 for the diagnosis and prognosis of AAA was further explored.

## Materials and methods

### Microarray assay

Utilizing the search terms “abdominal aortic aneurysm” and “Homo sapiens”, AAA transcriptome data were secured from the National Center for Biotechnology Information Gene Expression Omnibus (NCBI GEO) (http://www.ncbi.nlm.nih.gov/geo) [28]. The inclusion criteria were listed as follows: (1) AAA and non-AAA controls, (2) a sample size > 20, (3) non-Marfan atherosclerotic aortic aneurysms in the AAA group, (4) all data were available as free downloads and could be used for later assays. As per these criteria, three datasets (GSE57691, GSE98278, and GSE47472) were acquired, and their detailed information is provided in Supplementary Table S1. After removing the batch effect (BER) using the “Combat” function in the “sva” package, with default parameters, GSE57691 and GSE98278 were normalized and integrated using the “normalizeBetweenArrays” function as the training set. GSE47472 was utilized as an external validation set.

### Data processing and differentially expressed genes (DEGs) screening

The “limma” (v3.66.0) package was adopted to elucidate significant DEGs between the AAA and normal samples, with adjusted *P* < 0.05 and |log2FC|≥0.565 as the cut-off. log2FC ≥ 0.565 indicated up-regulated genes, and log2FC≤-0.565 signified down-regulated genes. 136 related genes to NETs were derived from a previous study (Supplementary Table S2) [29]. Then DEGs and NETs were intersected to identify DENETs for later assays.

### Functional enrichment analyses

Gene set enrichment analysis (GSEA) [30], Gene Ontology (GO) [31, 32], and Kyoto Encyclopedia of Genes and Genomes (KEGG) pathway analyses [33] were conducted to investigate the mechanism of DENETs in AAA. The R “ggplot2” [34], “GOplot” [35], and “enrichplot” packages were used for result visualization, respectively. In all three types of analyses, an adjusted P-value < 0.05 was indicative of statistical significance.

### Interaction network of DENETs

GeneMANIA (http://genemania.org) was adopted for predicting functionally similar genes in DENETs and their relationship with DENETs, encompassing protein-DNA and protein-protein interactions (PPI), pathways, and physiological and biochemical responses [36]. An interaction network of functionally similar genes in DENETs was constructed using GeneMANIA.

### Machine learning (ML) for signature gene screening

ML was also utilized to screen signature genes. Four ML algorithms (LASSO, RF, XGBoost, and Boruta) were adopted in this study. Specifically, LASSO assigns values to the coefficients of the variables to identify important genes. As an ensemble learning method based on the decision tree, RF assesses gene importance scores based on the Gini index. The XGBoost algorithm is implemented by the “xgboost” package, with max depth = 5 and eta = 0.3, and calculates importance scores based on gain scores [37]. Boruta performs full correlation feature selection using the “Boruta” package, with maxRuns = 500 as the stopping criterion, and performs an unsupervised clustering of data leveraging the K-Means algorithm [38]. The R “glmnet” (v4.1.10) [39] and “randomForest“(v4.7.1.2) packages were used for LASSO and RF, respectively. LASSO regression was conducted using the “cv.glmnet” function in the “glmnet” package to determine the minimum λ value by 10-fold cross-validation. By increasing decision trees, RF completed the final prediction by a majority vote [40]. The genes from XGBoost (v3.1.2.1) and Boruta (v9.0.0) were ranked by their importance. Finally, feature selection was performed by all ML models on the intersecting gene expression matrix in the training set. The intersecting genes from the four approaches were determined as potential signature genes.

### ROC curve analyses

The discriminatory potential of the signature genes was assessed by ROC curve analyses using the “pROC” package [41]. The area under the curve (AUC) was measured, and AUC > 0.65 was considered relatively good discriminatory power.

### Consensus clustering and immune infiltration analyses

Consensus clustering, an unsupervised clustering method [42], was employed to classify AAA samples into clusters based on DENETs. CIBERSORT was adopted for immune cell infiltration analysis in each clusters [43], with the iterations set to perm = 1000. The Spearman correlation analysis was executed on various types of immune cells across clusters. The R “ggpubr”, “vioplot” [44], “corrplot” [44], and “ggplot2” packages [34] were leveraged for result visualization.

### Gene set enrichment analysis (GSEA)

Based on the full gene expression matrix in the training set, the correlation of the target gene (IL6 or PADI4) with the expression of all other genes in the expression matrix was calculated by the Pearson’s correlation coefficient. This initially screened and identified biological pathways or functional modules that may co-vary with the target gene, which provided clues and hypotheses for subsequent mechanism studies. The included genes were all genes detected in the input expression matrix. With the expression of IL6 and PADI4 across all samples as “phenotype” labels, the rank correlation coefficients of all other genes with IL6 or PADI4 were calculated. Using the “clusterProfiler” R package (v4.18.2) [45], all genes were ranked based on the above correlation coefficients. Enrichment tests were performed on predefined gene sets (from GO and KEGG) in the Molecular Signatures Database (MSigDB) at the top or bottom of the ranking Tables [33, 46]. The enrichment score (ES) was calculated for each gene set and assessed for significance via 1000 label permutations, yielding normalized enrichment scores (NES) and false discovery rates (FDR). According to the original GSEA methodology [30], the significance threshold of the enrichment analysis was set to “pvalueCutoff = 0.05”, and multiple testing correction was conducted using pAdjustMethod="BH”. The “enrichplot” R package (v1.30.4) was utilized for visualizations of results.

### AAA mouse model by calcium chloride

After being weighed, male C57BL/6 mice (8–12 weeks old) were anesthetized utilizing sodium pentobarbital (60 mg/kg) by intraperitoneal injection. A median incision was made in the abdomen to expose the abdominal contents, and the abdominal aorta was dissected. The outer diameter of the aorta was measured from the midpoint between the opening of the left renal artery and the bifurcation of the iliac artery. A sterile cotton ball pre-soaked in 0.5 M CaCl_2_ solution was applied externally to this segment for 15 min, and a sterile gauze moistened with PBS was applied externally for 5 min and then removed. Then the abdominal cavity was washed with warm saline and closed [47]. In the normal group, 0.9% NaCl was used instead of CaCl_2_. All mice were weighed and sampled again two weeks postoperatively. Similar to the criteria in humans, an increase in diameter ≥ 50% of the normal value indicated AAA formation.

### Histological measurements

The abdominal aorta samples were fixed in 4% paraformaldehyde (Solarbio, China) for 24 h, dehydrated in 20% and 30% sucrose solution each for 1 d, embedded in OCT, and sliced into Sect.  (8 μm) using a freezing microtome (Leica CM1950). Following the cytoplasmic and nuclear staining using the HE staining kit (Solarbio, G1120-3*100 mL), the status of elastic fiber breakage was observed using the modified EVG staining kit (Solarbio, G1597-3*50 mL), and the intima calcified deposits were observed using the alizarin red staining kit (Beyotime, C0140-100 mL).

### Human sample collection

The diseased abdominal aortas from AAA patients in the Cardiovascular Surgery Department, ZhuJiang Hospital of Southern Medical University were harvested as clinical samples for this study. The study protocol was approved by the Medical Ethics Committee of ZhuJiang Hospital (Approval No.: 2023-KY-120- 02)), and all subjects gave their informed consent in writing. Besides, normal abdominal aortas from the specimen bank of Southern Medical University were harvested as the normal controls. Finally, five AAA samples and five normal samples were collected, and those used for Western blotting (WB) and quantitative polymerase chain reaction (qPCR) were snap-frozen with liquid nitrogen and immediately stored at -80 °C. The patient information is shown in the table below.

**Table 1:** Patient information for abdominal aorta samples

Characteristic	AAA (*n* = 5)	Normal (*n* = 5)
Age, median (range), Y	70(59–74)	69(61–73)
Sex, male	5(5)	5(5)
BMI (kg/m^2^)	24.97(± 1.86)	22.38(± 1.40)
Aortic diameter, median (range), mm	64(50–105)	NA
Smoking	3(5)	3(5)
Hypertension	4(5)	3(5)
Hyperlipidemia	3(5)	2(5)
Diabetes mellitus	2(5)	2(5)

*AAA* Abdominal aortic aneurysm, *NA* Not acquired

### RNA extraction and qPCR

Following the manufacturer’s instructions, total RNA was extracted from human abdominal aorta samples employing ISOGEN (Nippon Gene, Toyama, Japan). The cDNA synthesis kit (TaKaRa, Japan) was then used to transcribe the RNA into cDNA, and real-time qPCR was performed using SYBR Premix Ex Taq (TaKaRa, Japan). The relative changes in the expression of target genes were normalized to the expression of GAPDH by the 2^−ΔΔCt^ method. The primer sequences utilized in qPCR are listed in the table below:

**Table 2:** Primer sequences used in qPCR

Gene	Primer Sequence (5’-3’)
*GAPDH* (Homo sapiens)	Forward primer: GGAAGGTGAAGGTCGGAGTC Reverse primer: GTTGAGGTCAATGAAGGGGTC
*PADI4* (Homo sapiens)	Forward primer: GTTTAGGGTCAGACAGTCCTGG Reverse primer: AGATGTGAGTAGTGGCACATGC
*MMP9* (Homo sapiens)	Forward primer: CGGTTTGGAAACGCAGATGG Reverse primer: TGGGTGTAGAGTCTCTCGCT
*IL1β* (Homo sapiens)	Forward primer: CAGAAGTACCTGAGCTCGCC Reverse primer: CTGGAAGGAGCACTTCATCTGT
*IL6* (Homo sapiens)	Forward primer: GACCCAACCACAAATGCCAG Reverse primer: GTGCCCATGCTACATTTGCC
*TNF-α* (Homo sapiens)	Forward primer: CAAGGACAGCAGAGGACCAG Reverse primer: TGGCGTCTGAGGGTTGTTTT

### WB

The abdominal aorta samples were lysed by RIPA (Sigma Aldrich, USA), phosphatase inhibitors, and protease inhibitors (100:1:1), and proteins were extracted and quantified by BCA Protein Assay Kit (Thermo Scientific, USA). After boiling in a loading buffer for 5 min, the proteins were separated by 10% SDS-PAGE and transferred onto PVDF membranes. Then the membranes were blocked in 5% skim milk powder (Bio-Rad, USA) at room temperature for 2 h and washed with TBST (3 times × 5 min). Subsequently, they were incubated with primary antibodies against PADI4 (Abmart, TD6685S), MMP9 (Abcam, ab283575), MPO (R&D, AF3667), citrullinated histone H3 (CitH3) (Abcam, ab281584), IL6 (Wanleibio, WL02841), and GAPDH (Proteintech, 60004-1-Ig) at 4 °C overnight and with corresponding secondary antibodies at room temperature for 1 h. Labeled proteins were visualized by ECL (Bio-Rad, USA), and Quantity One 4.6.2 was adopted to normalize the band intensity to GAPDH.

### Immunofluorescence (IF) assay

After being blocked for 1 h at room temperature in PBS containing 3% bovine serum albumin, the abdominal aorta sections were incubated for 1 h at room temperature with primary antibodies against MPO and CitH3 (1:200) and corresponding Alexa Fluor-conjugated secondary antibodies (1:200). Then DAPI (Beyotime, China) was employed to stain the nuclei at room temperature for 5 min. A fluorescence microscope (Leica SP8, Germany) was leveraged to capture the fluorescence images.

### Statistical analysis

All data were described by mean ± standard deviation (SD) in independent experiments. GraphPad Prism 8 and R4.4.2 were utilized for statistical analysis. Comparison between two cohorts was executed by independent-samples t-test. The one-way ANOVA and least significant difference (LSD) post hoc test were utilized to compare multiple groups. *P* < 0.05 was indicative of statistical significance.

## Results

### DENETs Identified using “limma”

**Fig. 1:**
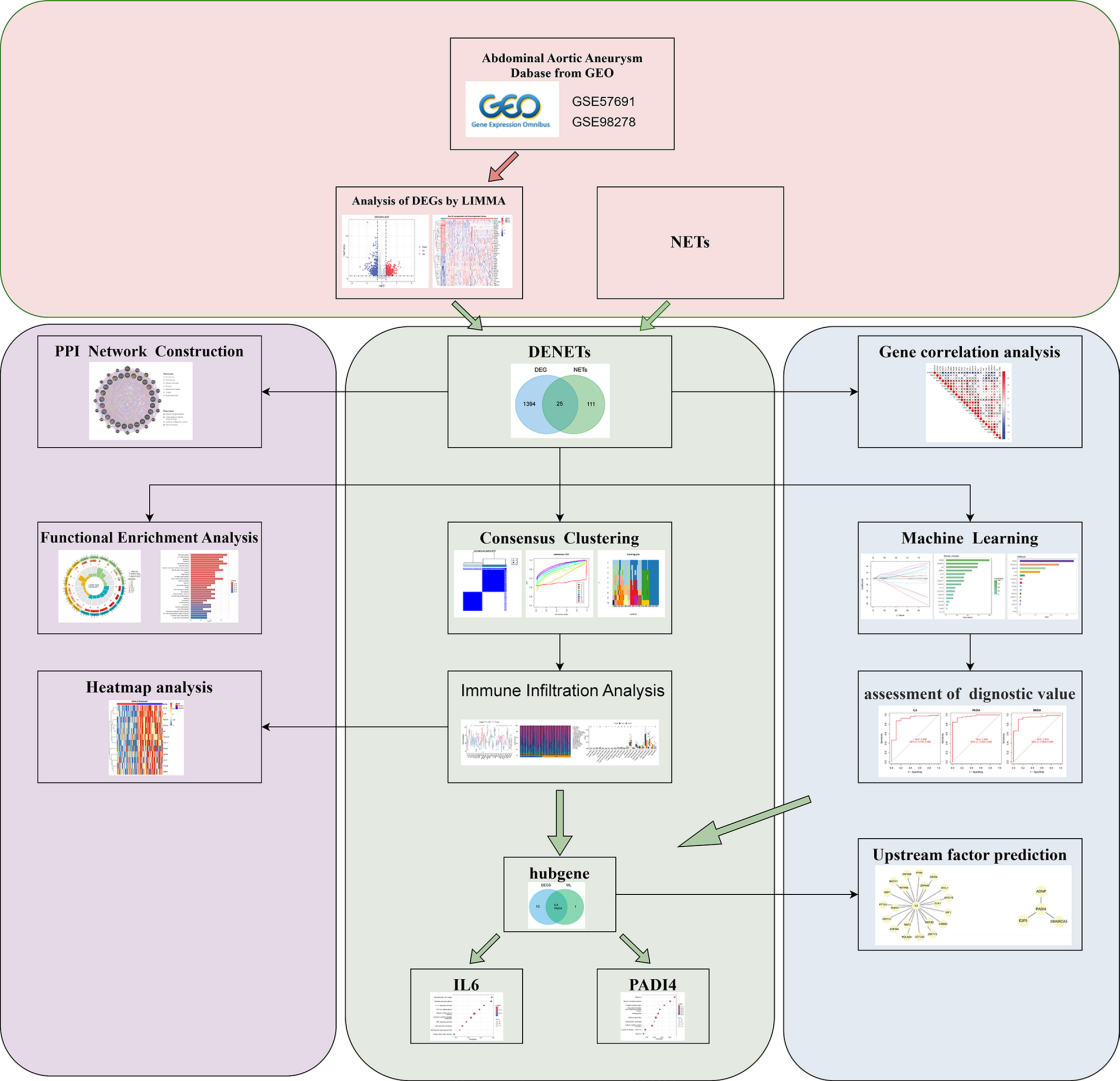
Flowchart for study design and multi-step analysis of bioinformatics data

**Fig. 2:**
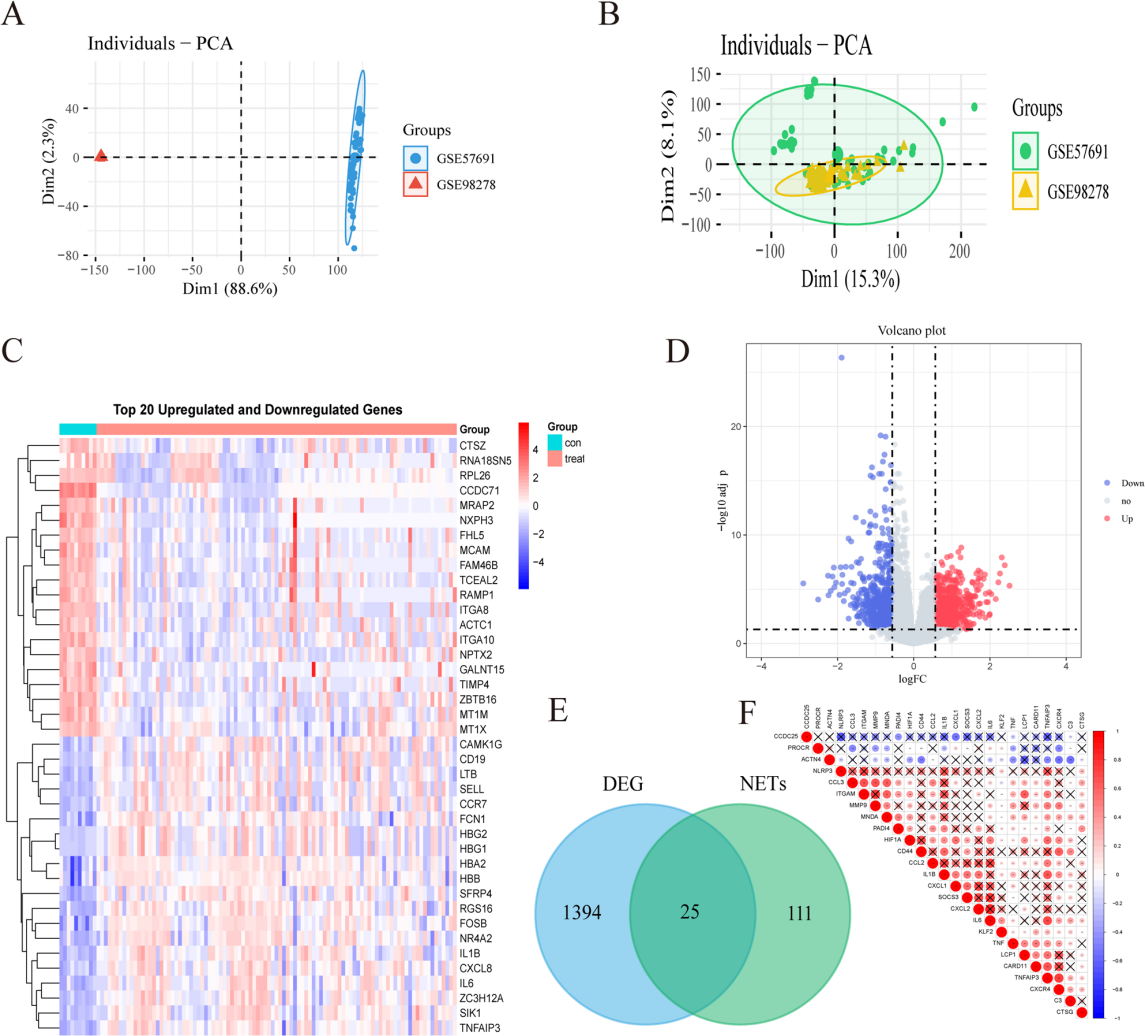
DEGs identified in AAA. **A** Pre-BER PCA. **B** Post**-**BER PCA. **C** Heatmap of the top 20 DEGs. **D** Volcano plot of DEGs (red: up-regulated, blue: down-regulated). **E** Venn diagram of DENETs. **F** Gene correlation of DENETs

The flowchart of this study is displayed in Fig. 1. After BER (Fig. 2A-B). The box plots after batch effect removal are shown in Supplementary Fig. S1(A) and S1(B). We obtained 1419 DEGs (Supplementary Table S3) from the intersected dataset based on the significance criteria, including 672 up-regulated (Supplementary Table S4) and 747 down-regulated ones (Supplementary Table S5), as depicted in the heatmap and volcano plot (Fig. 2C, D). DEGs were intersected with NETs to identify DENETs (Supplementary Table S6). The inter-gene correlation of DENETs (Fig. 2E) was analyzed utilizing the R “corrplot” package [44] (Fig. 2F).

### Functional enrichment analyses

**Fig. 3:**
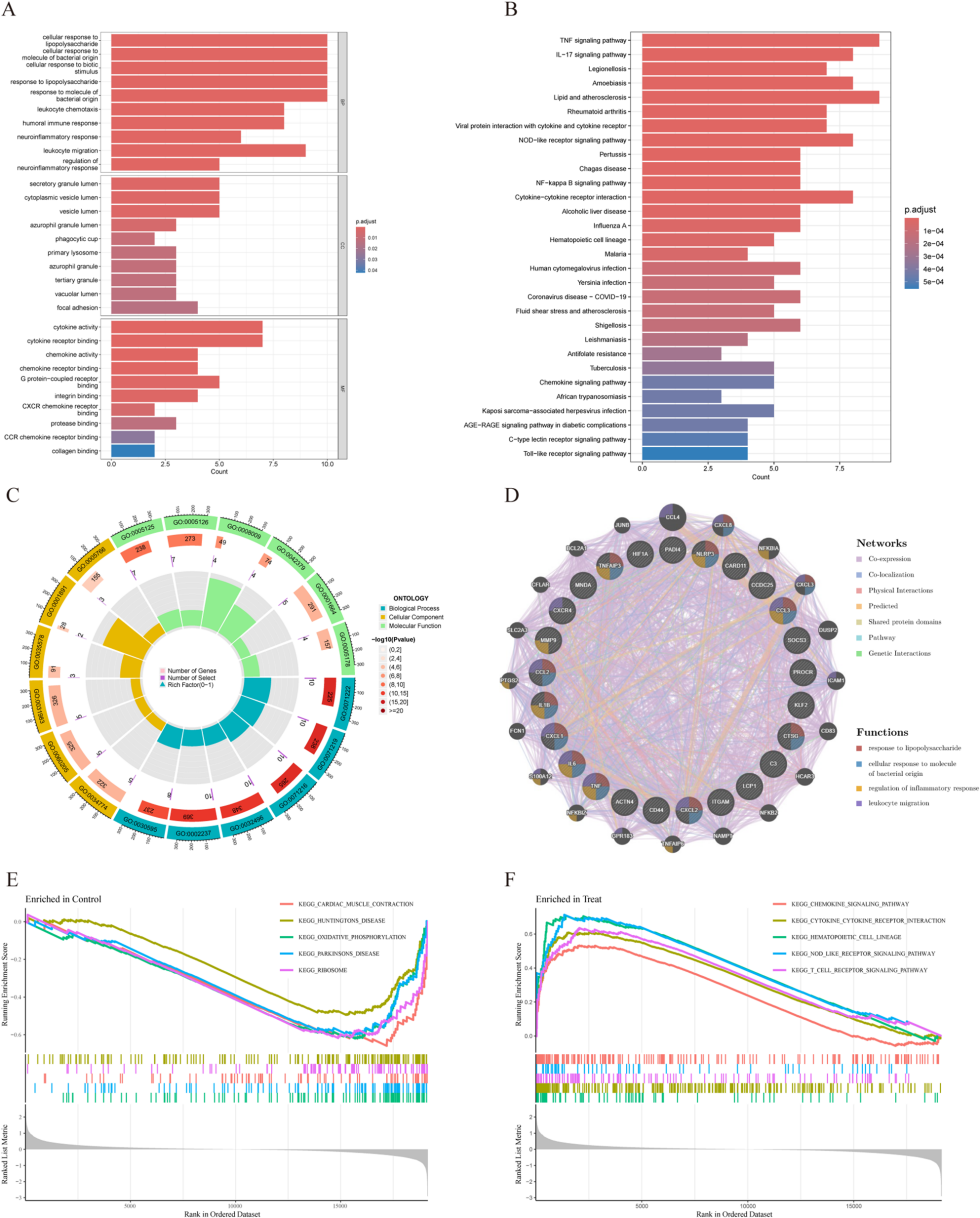
Functional enrichment analyses of DENETs. **A**,** C** Top 10 GO terms for biological process, cellular component, and molecular function in DENETs. The bar length reflects the enrichment score. **B** The top 30 KEGG pathways in DENETs. The X-axis and Y-axis denote the enrichment score of each pathway and the enriched pathway, respectively. **D** Interaction network of DENETs. **E**,** F** GSEA of DENETs: Down-regulated (**D**) and up-regulated (**F**) pathways

GSEA, GO, and KEGG functional enrichment analyses were implemented for DENETs (Fig. 3). The GO terms came from three domains: biological process (BP), cellular component (CC), and molecular function (MF) (Fig. 3A, C). The bubble plot for GO analysis is shown in Supplementary Fig. S1(C). For BP, DENETs were associated with cellular response to lipopolysaccharide, cellular response to molecule of bacterial origin, and cellular response to biotic stimulus. For CC, DENETs were highly enriched in secretory granule lumen, cytoplasmic vesicle lumen, and vesicle lumen. For MF, DENETs were mainly associated with cytokine activity, cytokine receptor binding, and G protein-coupled receptor binding. It can be seen that inflammation was an important player in AAA. Besides, KEGG analyses identified the TNF signaling pathway, Lipid and atherosclerosis, and IL17 signaling pathway as important pathways (Fig. 3B). The bubble plot for KEGG analysis is shown in Supplementary Fig. S1(D). A total of 20 functionally similar genes of DENETs were obtained using GeneMANIA (Fig. 3D), with the hub genes in the inner circle and the predicted genes in the outer circle. Consistent with previous findings, these genes mainly regulated the inflammatory response. The node colors indicated the protein functions, while the line colors represented the types of PPI. Besides, GSEA revealed that DENETs were predominantly enriched in the chemokine signaling pathway and cytokine-cytokine receptor interaction-related pathway (Fig. 3E, F). To sum up, DENETs were enriched in inflammation-associated pathways, which raised serum inflammatory marker levels [48] and increased the risk of chronic diseases [49].

### Signature genes screened by ML

**Fig. 4:**
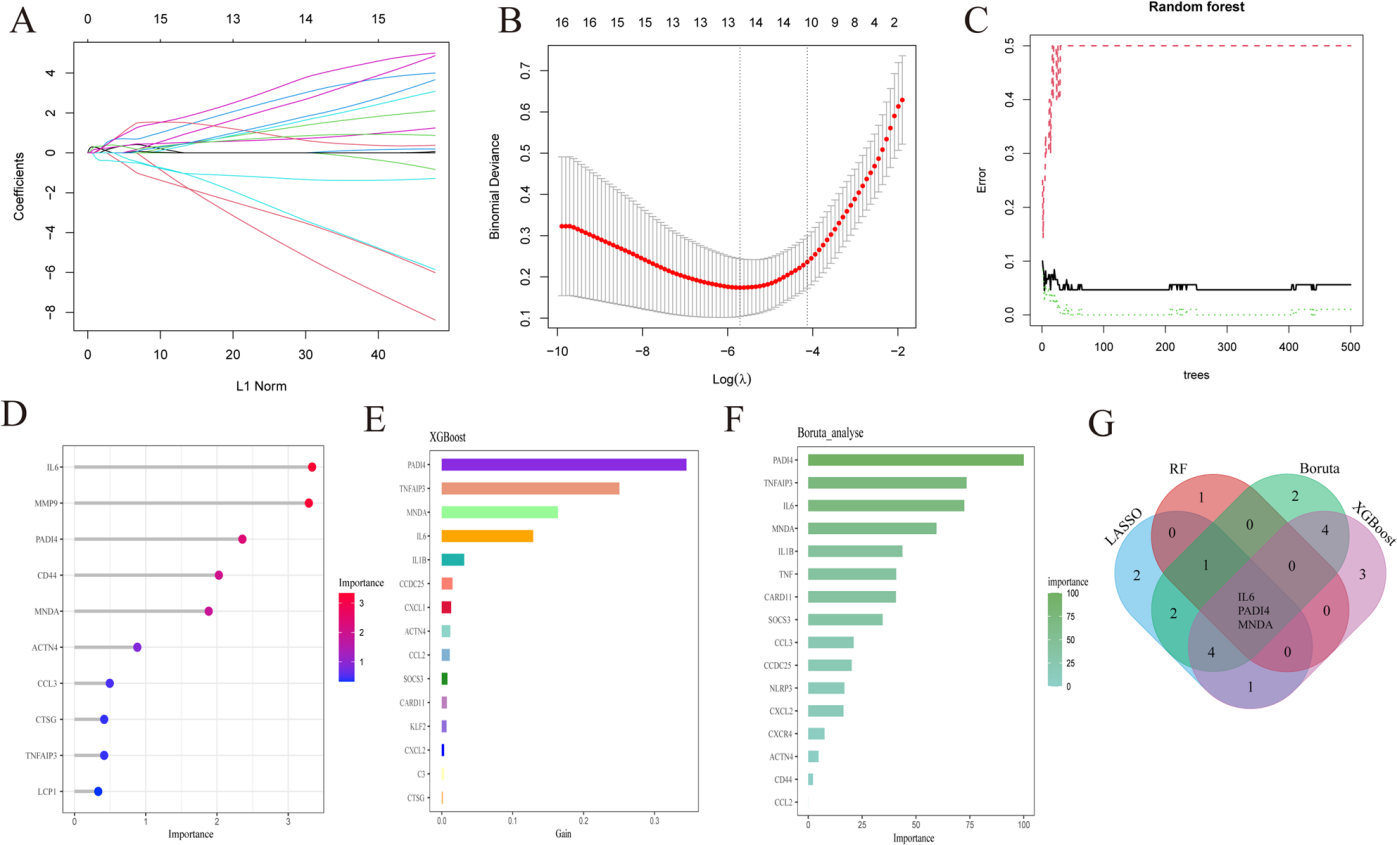
Signature genes screened by machine learning. **A** Path of regression coefficients. **B** Cross-validation curve. The X-axis and Y-axis represent the logλ of the penalty coefficient and the binomial deviance, respectively. The lower the value on the Y-axis, the better the fit. **C** Random forest plot. **D** Importance in random forest (VIP). As ranked by importance, the redder the color, the higher the importance. **E** XGBoost algorithm plot. **F** Boruta algorithm plot. **G** Venn diagram of signature gene screening by four ML methods

Signature genes were screened from the resulting 25 DENETs using four algorithms (LASSO, RF, XGBoost, and Boruta), and their intersection was taken. Based on the minimum Lambda (minimum λ = 0.003) LASSO (Fig. 4A, B), 13 genes (LCP1, CTSG, NLRP3, IL6, PROCR, CARD11, ACTN4, TNF, MNDA, IL1B, CD44, CCDC25, and PADI4) were screened. Five genes were obtained with Importance > 1 based on the RF (Fig. 4C, D). The top 15 and 16 genes from XGBoost and Boruta were screened as shown in Fig. 4E and F (Supplementary Table S7), respectively. Finally, three intersecting genes IL6, PADI4, and MNDA from the four methods were identified as potential signature genes for further evaluation Fig. 4G.

### Model validation

**Fig. 5:**
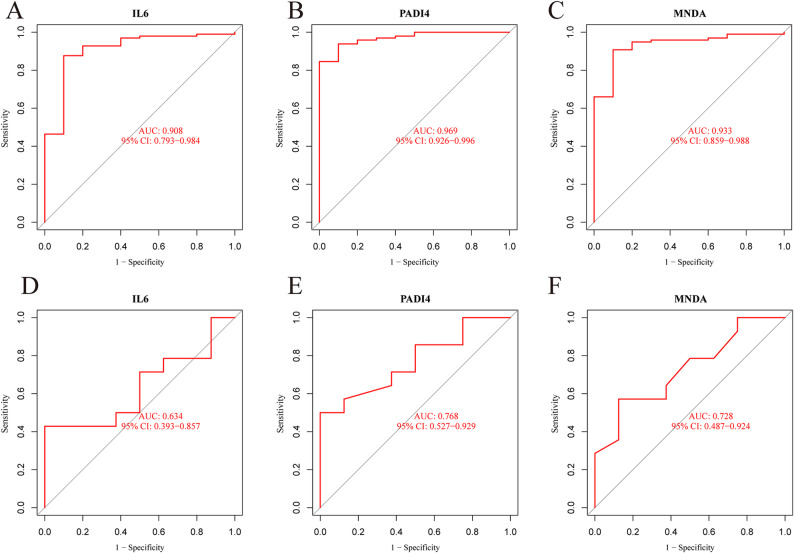
ROC analysis of identified signature genes. **A-C** ROC curves for signature genes in the internal validation set. The area under the red curve denotes AUC. **D-F** ROC curves for signature genes in the external validation set

The discriminatory potential of the signature genes was determined using ROC curve analyses. AUC was computed for each dataset. In the training set, the three genes exhibited relatively good discriminatory power between the AAA and normal groups (AUC > 0.65, Fig. 5A-C). External validation for ROC curves was conducted using GSE47472 (Fig. 5D-F). In summary, IL6, PADI4, and MNDA can serve as signature genes.

### Consensus clustering and immune infiltration analyses

**Fig. 6:**
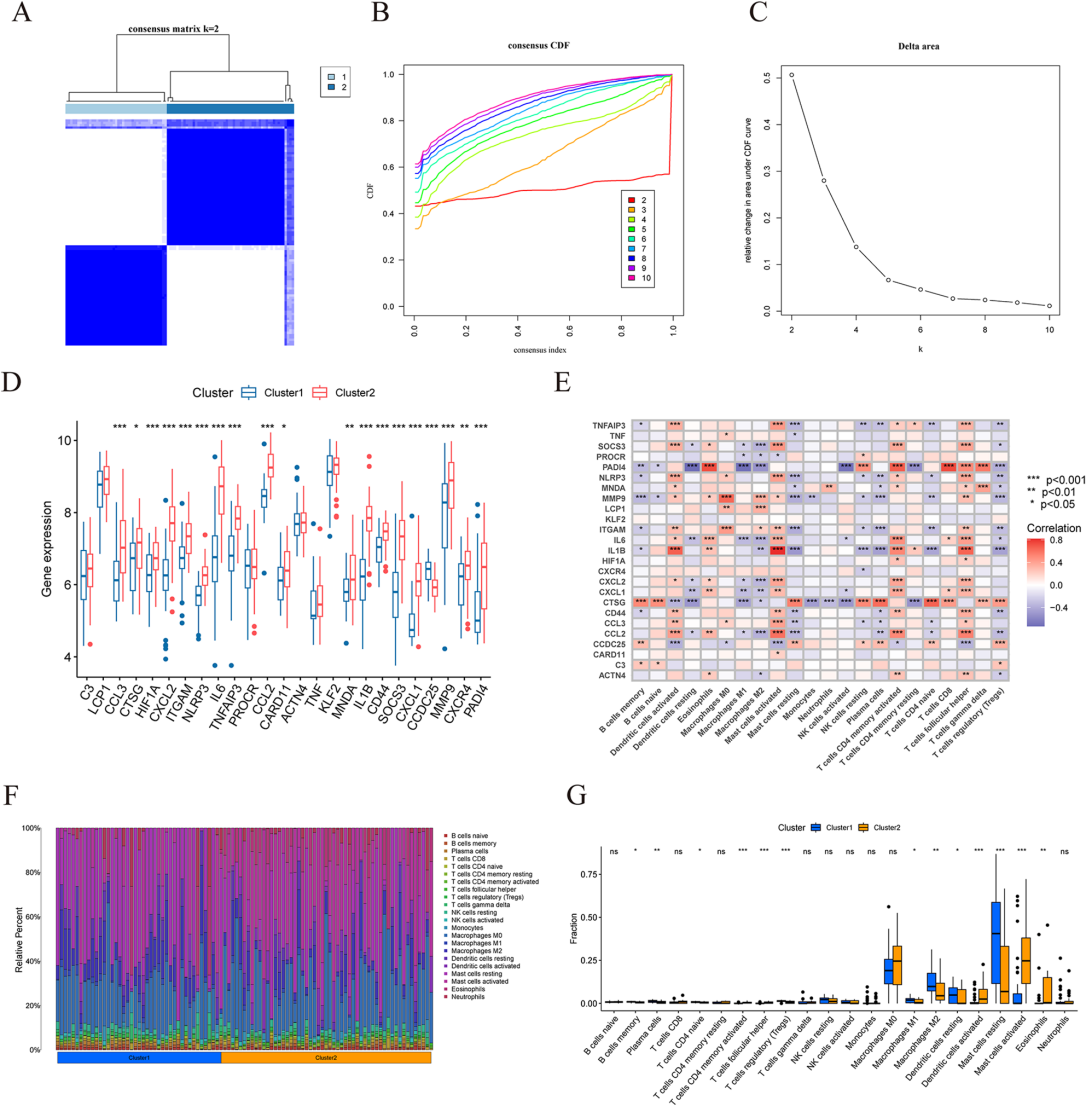
Consensus clustering and immune infiltration analyses. **A** Consensus matrix heatmap (k = 2). **B** Cumulative distribution function plot. **C** Delta area plot. **D** Box plot of gene expression (***P < 0.001, **P < 0.01, *P < 0.05). **E** Heatmap of immune cells-DENETs correlation (red: positive, gray: negative). The correlation is stronger when the color is darker. **F** Heatmap of immune cell infiltration. The X-axis and Y-axis show the sample classification and cell proportion, respectively. **G** Intergroup comparison of immune infiltration of 22 types of immune cells. ns: not significant; more * corresponds to more significant difference

To explore potential differences in the immune microenvironment across clusters in AAA, consensus clustering analysis was performed on DENETs. According to the consensus matrix heatmap, the consensus cumulative distribution function (CDF), and the Delta area plot, PAC yielded k = 2, and we observed that the CDF curve exhibited the gentlest decline when k = 2. The Delta area plot revealed a distinct inflection point, suggesting that the cluster result with k = 2 was the most robust and reliable. the optimal number of clusters (k = 2) was identified (Fig. 6A-C). AAA samples were classified into Cluster1 and Cluster2. The expression of 25 DENETs in AAA was compared between Cluster1 and Cluster2 (Fig. 6D). Inflammatory responses and immunoregulation are integral to AAA pathogenesis, and immune infiltration analysis can better reveal the immune effects on AAA. CIBERSORT was adopted to calculate the immune cell infiltration scores, and the correlation analysis was implemented on 22 types of immune cells with DENETs (Fig. 6E). The cell proportions in different samples and the degrees of immune cell infiltration in clusters are displayed in Fig. 6F and G, respectively. Compared with Cluster2, Cluster1 had more abundant infiltration of macrophages M1, macrophages M2, dendritic cells resting, and mast cells resting, and less infiltration of dendritic cells activated and mast cells activated. This indicated that M1 and M2 macrophages were more abundant in Cluster1. Moreover, strong interactions were observed between M2 (anti-inflammatory) or M1 (pro-inflammatory) macrophages and other immune cells that were key in the onset and progression of artery aneurysms [50]. Several studies have unraveled that phenotypic transformation disorders of macrophages in AAA may be linked to persistent elevations of serum inflammatory response [51]. To sum up, according to the correlations between infiltrating immune cells, the formation of NETs in AAA may have an association with inflammation and macrophage phenotypic transformation. In the future, functional experiments and larger-sample cohort studies should be combined to elucidate the specific mechanisms of different subtypes.

### GSEA of hub genes

**Fig. 7:**
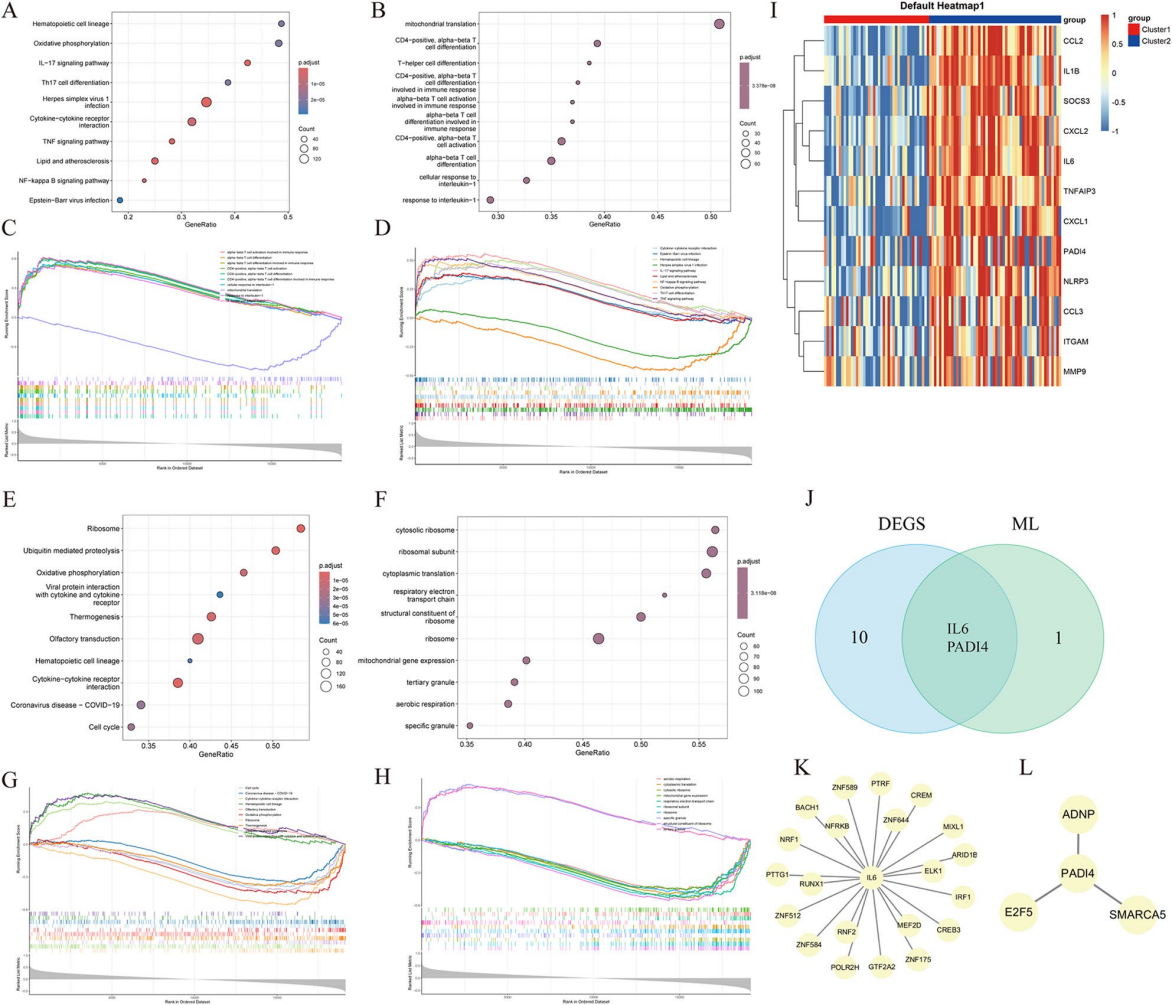
Single-sample GSEA and upstream transcription factor prediction. **A** IL6 KEGG diagram. **B** IL6 GO diagram. **C**,** D** IL6 GSEA plot. **E** PADI4 KEGG diagram. **F** PADI4 GO diagram.**G**,** H** PADI4 GSEA plot. **I** Heatmap of DEGs between Cluster1 and Cluster2. **J** Venn diagram of DEGs-ML intersecting genes. **K**,** L** IL6 and PADI4 upstream transcription factor prediction

GSEA results revealed that IL6 was probably correlated with inflammation and immune response, and PADI4 had possible associations with ribosomal oxidative phosphorylation and cytokine interactions (Fig. 7A-H). Twelve DEGs (IL1B, IL6, CCL2, CXCL2, SOCS3, TNFAIP3, CXCL1, NLRP3, ITGAM, CCL3, PADI4, and MMP9) were obtained between Cluster1 and Cluster2 in consensus clustering (Fig. 7I). Then, they were intersected with the signature genes screened by ML (Fig. 7J), and two intersecting hub genes IL6 and PADI4 were acquired. Considering the food diagnostic efficacy of the genes selected by ML algorithms and the intersecting genes selected by consensus clustering across various diseases and controls, as well as significant differences in the expression across different clusters, IL6 and PADI4 were ultimately selected.

### Upstream transcription factor prediction

NetworkAnalyst (http://www.networkanalyst.ca/faces/home.xhtml) is a gene expression profiling-based online visualization tool that can comprehensively analyze the functional pathways of hub genes and predict upstream transcription factors. The specific parameters are as follows: “Homo sapiens”, Official Gene Symbol, TF-gene interaction, ENCODE database, peak intensity signal < 500, and the predicted regulatory potential score < 1, with all other parameters default. Transcription factor-target gene regulatory relationships with adjusted P-values < 0.05 and composite confidence > 0.75 were preserved. The transcription factors included were provided by the database. We utilized NetworkAnalyst to construct the upstream transcription factor network, and visualized the results using Cytoscape (Fig. 7K and L). The exploratory analysis of transcription factors showed that BACH1, NRF1, and IRF1 might influence the IL6 expression by regulating redox equilibrium (e.g., Nrf2 pathway) or directly activating inflammatory signals (e.g., NF-κB and JAK-STAT pathways). SMARCA5 and E2F5 were possibly key upstream regulators of PADI4, which worked through chromatin remodeling and direct transcriptional activation, respectively. Further experimental validation is required to confirm the regulatory effects of these transcription factors on IL6 and PADI4.

### Validation of hub genes in AAA

**Fig. 8:**
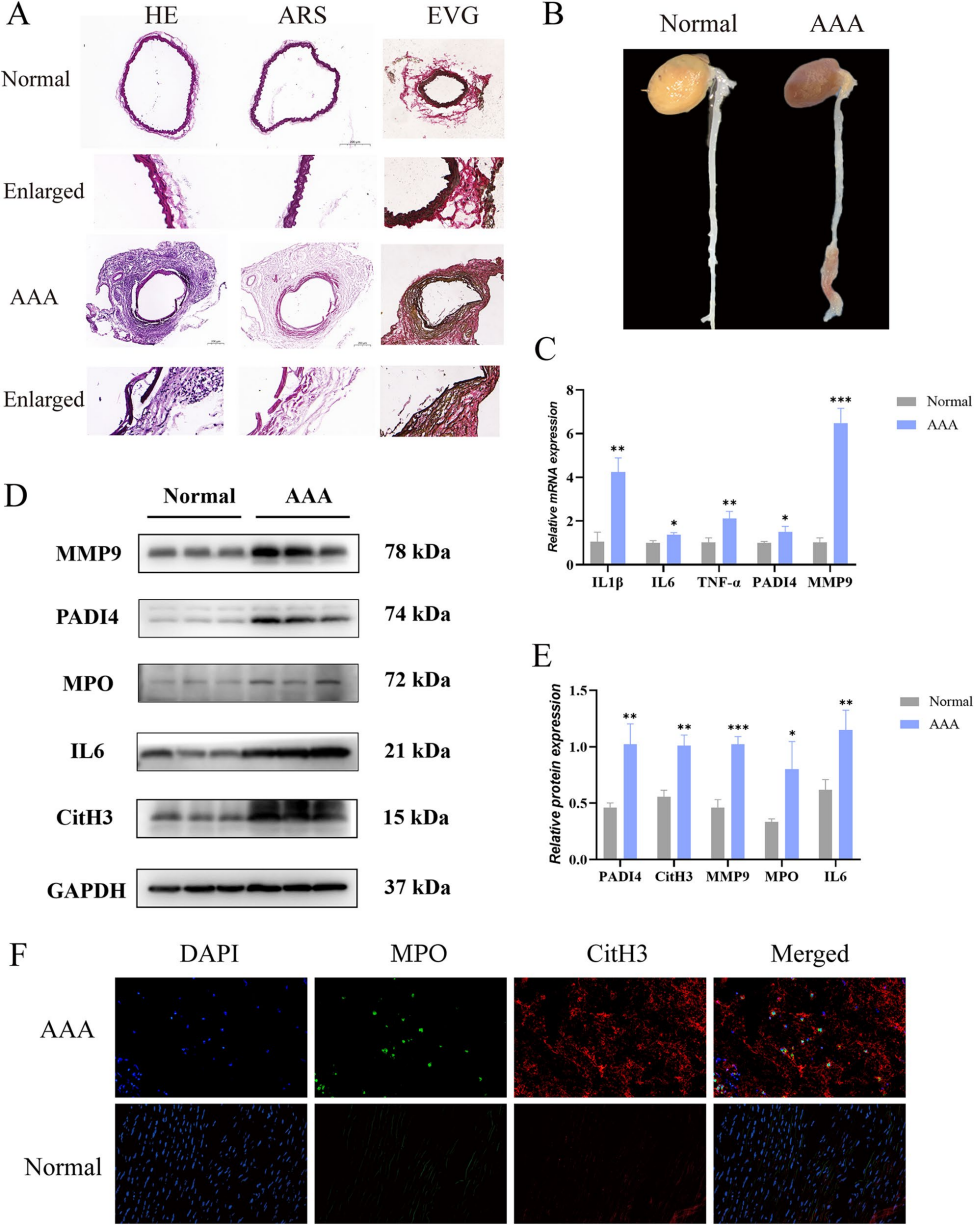
Expression of hub genes in aorta tissues. **A** Representative results of HE/alizarin red/EVG staining of abdominal aorta in CaCl_2_-modeled mice (scale bar = 200 μm, *n* = 5). **B** Naked-eye image of abdominal aortic enlargement in mice on Day 14 after modeling (*n* = 5). **C** Relative mRNA expressions of IL1β, IL6, TNF-α, PADI4, and MMP9 in human samples detected by qPCR (***P < 0.001, **P < 0.01, *P < 0.05, *n* = 3). **D** Representative protein bands in WB of MMP-9, PADI4, MPO, IL6, and CitH3 in human samples (*n* = 3). **E** Results of quantitative WB (***P < 0.001, **P < 0.01, *P < 0.05, *n* = 3). **F** Representative images of IF staining of aorta sections in human samples (scale bar = 100 μm, *n* = 3)

The hub genes were further validated by qPCR, WB, and IF. AAA was successfully induced in mice by applying calcium chloride around the aorta (Fig. 8B). The histological analysis of mouse abdominal aortic tissues confirmed key pathological features of AAA, such as structural damage (HE staining), severe elastic fiber breakage (EVG staining), and significant calcification (alizarin red staining) (Fig. 8A). Then the expressions of the hub genes identified and key NETs/inflammatory markers were quantified. The qPCR results revealed that the mRNA levels of IL6, PADI4, and MMP9 were significantly higher in human AAA tissue samples than in normal aortic tissues (Fig. 8C). This transcriptional activation was further validated at the protein level: The WB results showed that the protein expressions of IL6, PADI4, MMP9, and MPO significantly increased in human AAA tissue samples (Fig. 8D and E). More importantly, WB also revealed a marked increase in CitH3, a direct enzymatic product of PADI4 and a typical marker of NETs. Furthermore, IF co-staining was performed and it was found that a large number of extracellular structures were present and positive for both MPO and CitH3 (Fig. 8F). This colocalization constitutes a definite histological feature of NETs. These structures of NETs were predominantly localized in regions with intense inflammatory infiltration within the aneurysm wall. To sum up, these findings indicate that IL6 and PADI4 play central roles in driving the NETs-induced inflammatory response in AAA.

## Discussion

Potential hub genes in AAA have been studied previously. For example, Ouyang et al. revealed the role of mesenchymal stem cell (MSC)-derived nicotinamide phosphoribosyltransferase (NAMPT) as a potential treatment for AAA in a transcriptome analysis [52]. Teng et al. carried out a weighted gene co-expression network analysis (WGCNA) to determine MEDAG and SERPINE1 as genes involved in hypoxia and rupture in AAA [53]. Zhang et al. Have also found by WGCNA that MMP16, MMP17, Ctsa, Ctsc, and Ctsw are implicated in ECM degradation in AAA [54]. In this study, we screened the signature genes by ML and validated the model performance using ROC curves in GSE47472, followed by clustering and immune infiltration analyses. Finally, two hub genes (IL6 and PADI4) of NETs in the pathogenesis of AAA were identified, and their differential expression was further verified by qPCR, WB, and IF, which greatly enhanced the robustness of our findings and helped further study the mechanism underlying NETs in the formation and development of AAA.

Smooth muscle cell degeneration, ECM degradation, and persistent inflammatory infiltration are the typical pathological features of AAA. The inflammatory process plays a crucial role in AAA and significantly influences aortic remodeling. Macrophages are closely linked to inflammation, and they generate chemokines and cytokines in response to tissue damage, thereby altering the microenvironment in the AAA wall. Meanwhile, macrophages also exert both pathogenic and repair effects by participating in ECM remodeling, promoting and resolving inflammation, and facilitating tissue healing [50]. NETs are not only a pivotal defense mechanism of innate immunity against pathogens [55] but also are associated with cancer [56], autoimmune diseases, and chronic inflammation [57, 58]. Moreover, NETs can induce pro-inflammatory immune responses by activating endothelial cells, antigen-presenting cells, and platelets [23, 27], and cause atherosclerosis by promoting plaque formation and arterial thrombosis.

The formation of NETs relies on histone citrullination mediated by PADI4 [59]. When neutrophils are activated by reactive oxygen species and cytokines in the AAA microenvironment, PADI4 will be recruited to the nucleus. PADI4 is an enzyme with a high expression in myeloid cells (especially neutrophils), and its core function is to catalyze the citrullination of arginine residues in proteins such as histone H3/H2A/H4 [60]. This modification can neutralize histone charges and contribute to chromatin depolymerization, laying a foundation for the release of NETs [59]. First discovered in human leukemia HL-60 cells [61], PADI4 (also known as PAD4/PADV) is abundantly expressed in peripheral blood neutrophils [62, 63]. PADI4 activation and elevated citrullinated proteins have been verified in inflammatory diseases such as rheumatoid arthritis, which can induce the production of anti-citrullinated peptide antibodies (ACPA) and the release of TNF-α, triggering an inflammatory response [64]. Stimulated neutrophils contain citrullinated vimentin, and autoantibodies against citrullinated vimentin can cause the formation of NETs [65]. With the formation of NETs, more ACPA is generated, leading to the production of IL6, IL8, chemokines, and adhesion proteins [66]. Meanwhile, IL6 produced by infiltrating macrophages, lymphocytes, and vascular cells further synergistically drives the inflammatory network.

**Fig. 9:**
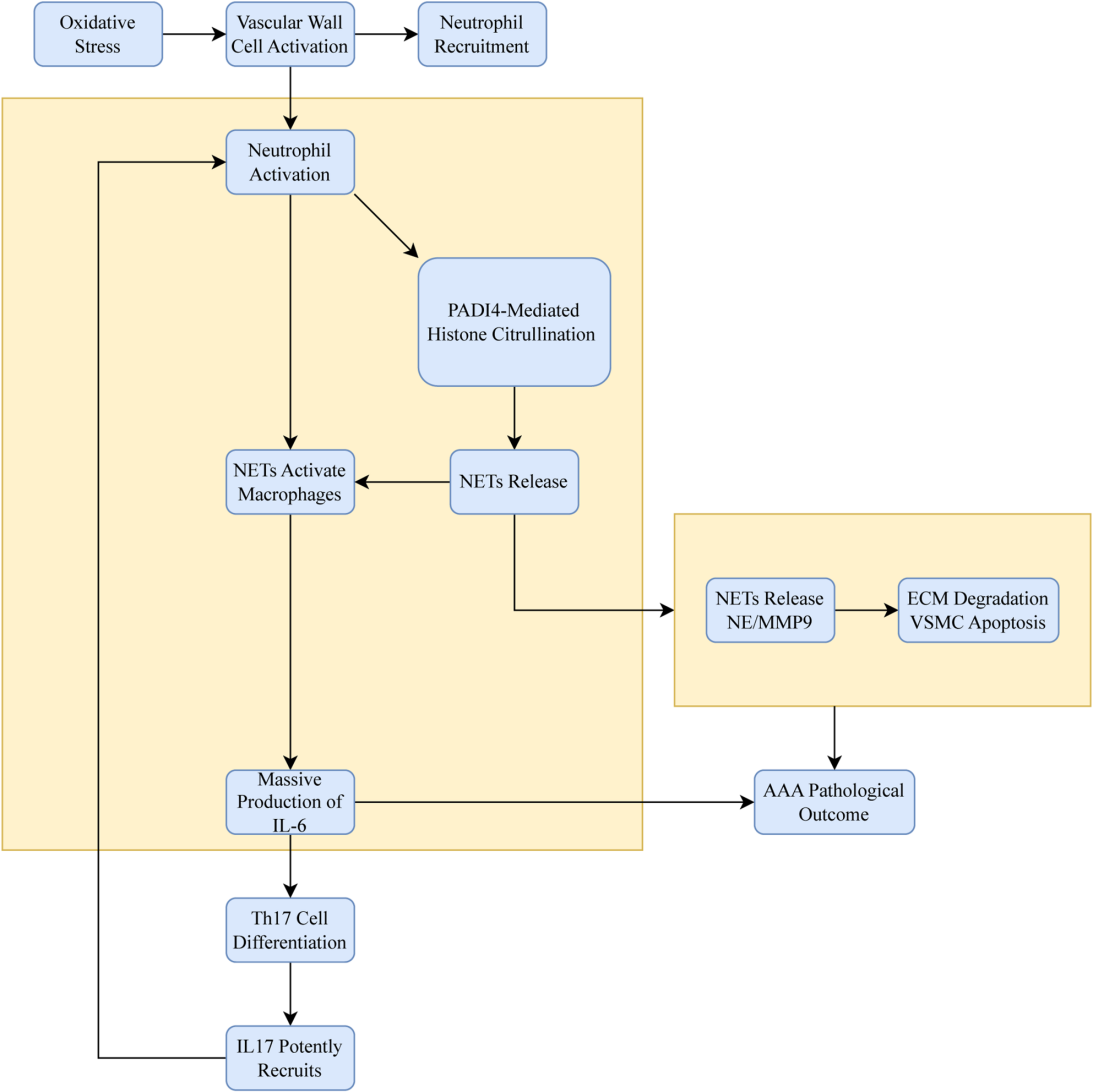
Relationship diagram of IL6, PADI4 and NETs

IL6, a pleiotropic pro-inflammatory cytokine, is primarily produced in AAA by infiltrating macrophages, lymphocytes, and activated vascular cells (e.g., endothelial cells, smooth muscle cells). IL6 can act on neutrophils through its classical pathway (membrane receptor IL6R/gp130) to activate oxidative responses, creating favorable conditions for NETs formation. Moreover, IL6 also forms a powerful inflammatory network with other key factors (e.g., IL-1β, TNF-α) in the AAA microenvironment, significantly promoting NETs production. Histone and DNA components on NETs can be recognized by Toll-like receptors on macrophages and dendritic cells, which significantly trigger the generation of cytokines, such as IL6, IL-1β, and TNF-α [57]. By promoting the production of IL6, NETs drive differentiation of CD4 + T cells into pro-inflammatory Th17 cells. Th17 cells secrete IL-17 to further recruit and activate neutrophils, creating another positive feedback loop. NE and MMP9 on NETs can directly degrade the ECM and induce apoptosis of vascular smooth muscle cells. During this process, IL6 can enhance cell sensitivity to these stimuli. The roles of IL6 and PADI4 in NETs formation and AAA pathogenesis are systematically elucidated in Fig. 9. The immune infiltration analysis revealed the different degrees of immune cell infiltration across clusters, especially with more abundant infiltration of M1 and M2 macrophages. This suggests that the development of aneurysms may be associated with the NETs-induced inflammatory responses and M2 (anti-inflammatory)/M1 (pro-inflammatory) conversion of macrophages, leading to phenotypic modulation of smooth muscle cells. To sum up, PADI4 can modulate inflammatory responses via NETs and may serve as a novel therapeutic target for AAA in the future.

This study is innovative in that it identifies new targets for NETs in AAA for the first, providing new ideas for the AAA treatment. However, some limitations are worth noting. First, the ability to collect samples is restricted due to the advent of interventional procedures, so the number of human abdominal aorta samples harvested was smaller, which may affect the precision and reliability of our findings. Second, sample imbalance in the dataset remains a limitation, and a larger sample size is needed to validate these findings in the future. The limited number of CIRBERSORT samples compromised analytical accuracy, so more samples should be included in future studies. In addition, the AUC threshold of > 0.65 used in this study was applicable only to preliminary screening, with limited diagnostic efficacy. In the future, combined with expression data from serum samples, large-scale, multicenter cohorts are needed to determine the diagnostic efficacy of the identified hub genes.

## Conclusion

In conclusion, the extensive bioinformatics analysis identifies and validates that IL6 and PADI4 play a pivotal in AAA by NETs. The findings furnish new targets and insights for the management of AAA.

## Supplementary Information

Supplementary Material 1.

Supplementary Material 2.

Supplementary Material 3.

Supplementary Material 4.

Supplementary Material 5.

Supplementary Material 6.

Supplementary Material 7.

Supplementary Material 8.

## Data Availability

The data are available upon request.

## Abbreviations

AAA: Abdominal aortic aneurysm
NETs: Neutrophil extracellular traps
IL6: Interleukin-6
PADI4: Peptidylarginine deiminase 4
VSMCs: Vascular smooth muscle cells
MPO: Myeloperoxidase
MMP: Matrix metalloproteinase
NE: Neutrophil elastase
ECM: Extracellular matrix
KEGG: Kyoto Encyclopedia of Genes and Genomes
CitH3: Citrullinated histone
NAMPT: Nicotinamide phosphoribosyltransferase
WGCNA: Weighted gene co-expression network analysis
ACPA: Anti-citrullinated peptide antibodies
