# Supplementary figures and images for: Hub genes of neutrophil extracellular traps in abdominal aortic aneurysm: a bioinformatics analysis
Source: Hereditas. 2026 Apr 20;163:69. doi: 10.1186/s41065-026-00663-0 (PMC13227812; doi:10.1186/s41065-026-00663-0)

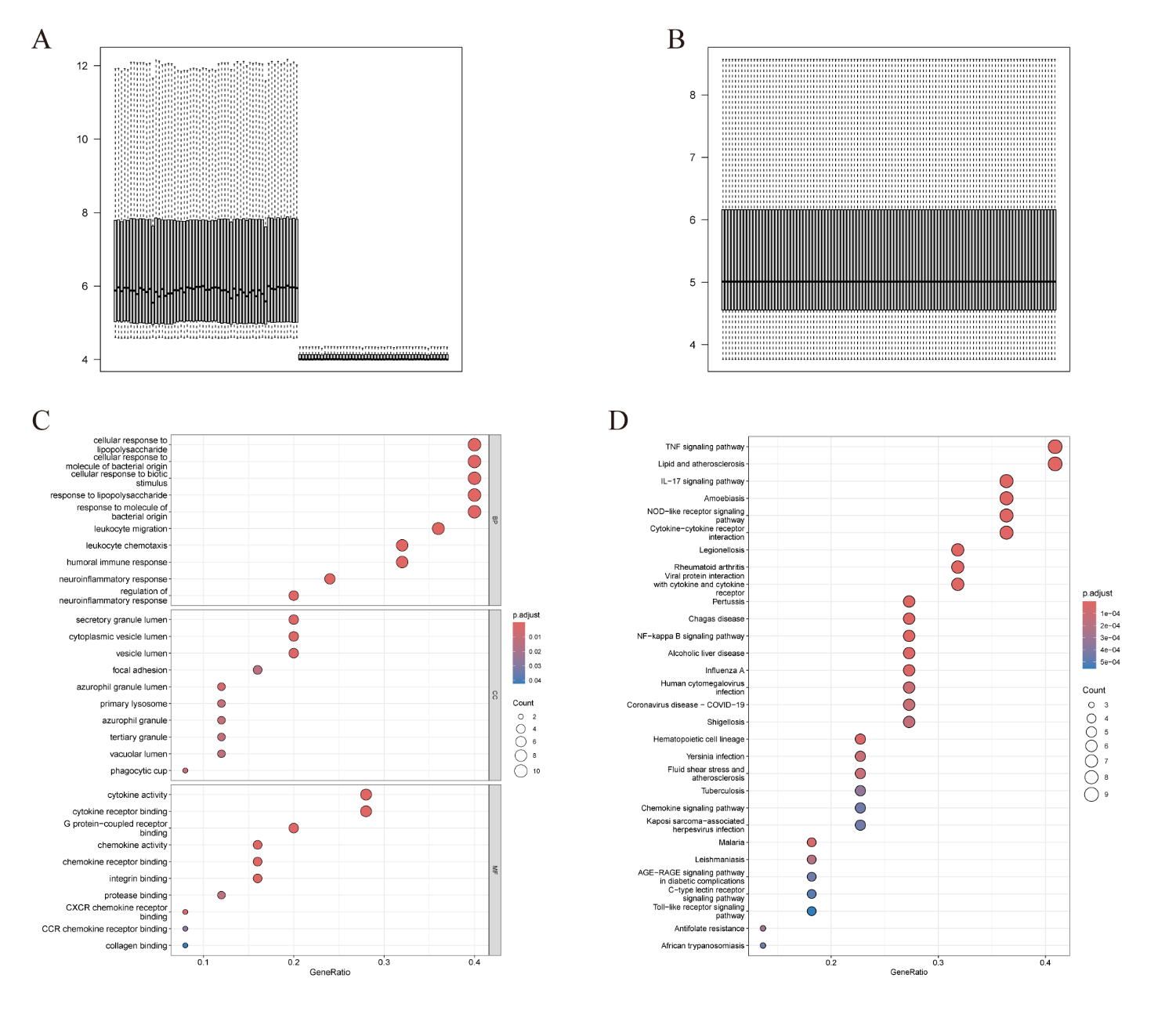

Supplement: Supplementary file 1 — Supplementary Material 1. [file 41065_2026_663_MOESM1_ESM.tif]
